# The Monothiol Glutaredoxin Grx4 Influences Iron Homeostasis and Virulence in *Ustilago maydis*

**DOI:** 10.3390/jof9111112

**Published:** 2023-11-17

**Authors:** Sean W. McCotter, Matthias Kretschmer, Christopher W. J. Lee, Kai Heimel, James W. Kronstad

**Affiliations:** 1 Michael Smith Laboratories, Department of Microbiology and Immunology, University of British Columbia, 301-2185 East Mall, Vancouver, BC V6T 1Z4, Canada; swmccotter@gmail.com (S.W.M.); kretschm@msl.ubc.ca (M.K.); christopherwjlee@msl.ubc.ca (C.W.J.L.); 2 Institute of Microbiology and Genetics, Department of Microbial Cell Biology, Göttingen Center for Molecular Biosciences (GZMB), University of Göttingen, Grisebachstr. 8, D-37077 Göttingen, Germany

**Keywords:** melanin, siderophore, infection, corn smut

## Abstract

The corn smut fungus, *Ustilago maydis*, is an excellent model for studying biotrophic plant-pathogen interactions, including nutritional adaptation to the host environment. Iron acquisition during host colonization is a key aspect of microbial pathogenesis yet less is known about this process for fungal pathogens of plants. Monothiol glutaredoxins are central regulators of key cellular functions in fungi, including iron homeostasis, cell wall integrity, and redox status via interactions with transcription factors, iron-sulfur clusters, and glutathione. In this study, the roles of the monothiol glutaredoxin Grx4 in the biology of *U. maydis* were investigated by constructing strains expressing a conditional allele of *grx4* under the control of the arabinose-inducible, glucose-repressible promoter *P_crg_*_1_. The use of conditional expression was necessary because Grx4 appeared to be essential for *U. maydis.* Transcriptome and genetic analyses with strains depleted in Grx4 revealed that the protein participates in the regulation of iron acquisition functions and is necessary for the ability of *U. maydis* to cause disease on maize seedlings. Taken together, this study supports the growing appreciation of monothiol glutaredoxins as key regulators of virulence-related phenotypes in pathogenic fungi.

## 1. Introduction

The pathogenic fungus *Ustilago maydis* causes corn smut disease on maize (*Zea mays*) and its ancestor teosinte [1,2]. The fungus induces chlorotic tumors on all aerial parts of the host plant, and the tumors become filled with masses of melanized resting spores (teliospores) that facilitate the dormancy and dissemination of the fungus. *U. maydis* has emerged as a useful model to investigate mechanisms of fungal biotrophy, including adaptation to the host nutritional environment and the activities of a plethora of effector proteins delivered during colonization [2,3,4,5]. The utility of *U. maydis* as a model is particularly important because many biotrophic plant pathogens, such as the rust fungi, cause devastating crop losses but are difficult to study in the laboratory. Haploid strains of *U. maydis* demonstrate yeast-like growth in axenic culture, facilitating experimentation [2,4].

Monothiol glutaredoxins such as Grx4 are key regulators of iron homeostasis in fungi and communicate the iron status of the cell to transcription factors that regulate iron uptake functions [6]. For example, foundational work established that the Grx3/4 proteins interact with the iron responsive transcription factor Aft1 in *Saccharomyces cerevisiae* [7]. Subsequent studies demonstrated similar regulatory influences for monothiol glutaredoxins with the iron regulators in *Schizosaccharomyces pombe* (Fep1) [8], *Cryptococcus neoformans* (Cir1) [9], and *Aspergillus fumigatus* (SreA) [10]. Grx4 in *C. neoformans* provides a particularly informative example. This basidiomycete is relatively closely related to *U. maydis* in comparison with the other fungi mentioned, which are ascomycetes. *C. neoformans* strains harboring a disrupted allele of *grx4* are avirulent in a murine model of cryptococcosis and demonstrate deficiencies in key virulence factors, such as melanin, growth at 37 °C and 39 °C, and elaboration of the polysaccharide capsule. The *grx4*-disrupted strains also showed increased sensitivity to ROS stress, cell wall stress, and DNA-damaging agents, along with mitochondrial dysfunction [9,11]. Additionally, the *grx4*-disrupted strains demonstrated susceptibility to iron starvation and iron chelation, indicating defects in iron sensing and uptake. The physical interaction between Grx4 and Cir1 was confirmed via yeast-two-hybrid analysis [9]. The iron responsive transcription factor of *U. maydis*, Urbs1, is an ortholog of Cir1 from *C. neoformans*. Urbs1 regulates the expression of the high-affinity iron uptake system encoded by *fer1* and *fer2* and the siderophore system encoded by the *fer3*-*fer10*, *sid1*, and *sid2* genes [12,13,14,15]. 

Given the close sequence similarity between Urbs1 and other fungal iron regulators that interact with Grx4, we hypothesized that Grx4 (encoded by UMAG_04223) is important for iron homeostasis in *U. maydis*. Therefore, we used conditional *grx4* mutants in this study to investigate the contribution of Grx4 to the regulation of iron homeostasis and virulence. As in *A. fumigatus*, Grx4 was found to be essential in *U. maydis* [10]. Conditional mutants allowed for an evaluation of the impact of Grx4 depletion on the transcriptome. This analysis confirmed the anticipated contribution of Grx4 to iron homeostasis, including regulation of the high-affinity iron uptake system encoded by *fer1* and *fer2* that contributes to virulence in *U. maydis* [13]. In this context, our analysis revealed that Grx4 plays a role in biotrophy because mis-regulation of Grx4 resulted in attenuated virulence in maize seedlings, especially for the prominent symptom of tumor formation.

## 2. Materials and Methods

### 2.1. Growth Conditions

Wild type (wt) *U. maydis* strains FB1, FB2, and derived independent conditional strains (*P_crg1_::grx4*) were routinely grown on PDA plates and in broth (PDB) plus 1% arabinose in the case of the conditional mutants. Cells were stored at −80 °C at a final concentration of 30% glycerol with PDB for the wt strains and with 1% arabinose for the mutant strains. Mating was routinely tested on double complete medium (DCM) agar with activated charcoal and 1% glucose or 1% arabinose. Carbon sources and chemicals were obtained from Sigma-Aldrich (Oakville, ON, Canada).

### 2.2. Construction of P_crg1_::grx4 in Strains FB1 and FB2

Deletion or disruption of *grx4* in *U. maydis* was unsuccessful, suggesting that Grx4 is essential for growth. Thus, a regulated strain approach was adopted and *P_crg1_::grx4* mutants were constructed via the following strategy. The *grx4* gene was amplified from FB2 genomic DNA using primers 276 and 277 (primers are listed in Table S1). A total of 1.0 kb of the sequence upstream of the *grx4* start codon was amplified with primers 280 and 281. The vector backbone of plasmid pMF2-1n was amplified using primers 282 and 279. The nourseothricin resistance cassette and the arabinose-inducible promoter *P_crg1_* were amplified from pMF2-1n with the primers 283 and 278 [16]. The PCR products were DpnI digested and cloned with a one-step method using FastCloning [17] into *Escherichia coli* DH5α chemically competent cells. The resulting plasmid pSWM-02 was used to amplify the 4.8 kb *P_crg1_::grx4* construct with primers 284 and 285. The construct was integrated into the *grx4* native locus of strains FB1 and FB2 by protoplast-mediated transformation [18]. The conditional strains were selected with 150 µg mL^−1^ nourseothricin and 1% arabinose. Correct genomic integration was confirmed with PCR, genomic hybridization [19], a PCR RFLP analysis, and by sequencing of the integration site. The constructed and verified strains are listed in Table S2.

### 2.3. Grx4 Depletion

Cells were grown overnight in test tubes with 5 mL PDB; 1% arabinose was included in the mutant cultures because this condition was found to allow growth. The cells were collected and washed twice with 5 mL minimal medium (MM) without a carbon source [1]. Cells were resuspended in 1 mL MM and transferred to test tubes with 5 mL MM plus 1% glucose to deplete Grx4 for 24 h. The MM cultures with initial pH of 7.0 were incubated at 30 °C in an orbital shaker with 220 rpm. After 24 h, the cells were harvested and washed twice with MM without a carbon source. Routinely, cells were counted and used at 10^6^ cells for inoculation in 5 mL MM in test tubes with either 1% glucose or 1% arabinose. The cultures were incubated for 72 h at 30 °C in an orbital shaker with 220 rpm. Cell numbers were determined at 24 h, 48 h and 78 h. Other growth conditions and additions of chemicals or carbon sources are indicated in the text.

### 2.4. Low Iron Minimal Medium (MM)

Regular MM contains a low level of FeCl_3_ (0.185 μM). To test strains for their iron response, low iron MM, which is free from any source of iron, was created by chelating iron from milliQ water with Chelex. The salt solution was prepared without FeCl_3_. All other components of the low iron MM are identical to regular MM [1,5]. Glucose and arabinose were used at 1%. Pre-starvation for iron and Grx4 depletion were performed in low iron MM for 24 h with 1% glucose, similar to as described above for Grx4 depletion. FeCl_3_ was added at 50 μM and heme at 30 μM to assess dependence on iron excess for mutant growth.

### 2.5. Cell Size Measurements

The size of cells for the wt and *P_crg1_::grx4* strains was measured microscopically for 25 cells for each biological replicate for cells grown in PDB (wt), PDB plus 1% arabinose (conditional mutants), and at the 24 h Grx4 depletion stage in MM plus 1% glucose (wt and conditional mutants). The average length for cells from each biological repeat was determined and the biological repeats were averaged. Calcofluor white and DAPI staining were performed to investigate the number of cell compartments and the number of nuclei per cell compartment. Calcofluor staining was performed as follows: One drop of a solution of calcofluor white (1 g/L, Sigma Aldrich, Oakville, ON, Canada) was added to a drop of cell culture on a glass slide. After placement of a cover slip, the cells were stained for 1 min prior to microscopic examination under UV light (λEx = 355 nm) at 100× to 400× magnification. Nuclear staining was performed using ProLong Gold antifade with DAPI (Invitrogen, Thermo Fisher, Burlington, ON, Canada), and the solution was added directly to cell samples. Subsequent microscopy was performed as for calcofluor white staining.

### 2.6. RNA Isolation, RNA-Seq Analysis, and Quantitative PCR

For the FB1 and corresponding *P_crg1_::grx4* strains, cells were grown overnight in 5 mL PDB (wt) plus 1% arabinose for the mutants. The cells were collected and washed twice with 5 mL MM without a carbon source. Cells were resuspended in 1 mL MM and transferred to 5 mL MM with 1% glucose to deplete Grx4 for 24 h. After 24 h, all cells were harvested and washed twice with MM without a carbon source. Cells were resuspended in 1 mL MM and divided into two aliquots resulting in ~10^8^ cells to be inoculated for each 5 mL of MM with 1% glucose or 5 mL of MM with 1% arabinose. Cells were collected after 1 h or 5 h. For the 24 h timepoint, cells were prepared as described before; however, FB1 5 × 10^6^ cells and 5 × 10^7^ cells were incubated in 5 mL MM with 1% glucose and 1% arabinose, respectively. For the mutant, 5 × 10^7^ cells for both 5 mL of MM with 1% glucose or 1% arabinose were used. For the 48 h timepoint, cells were prepared as described before; however, FB1 2.5 × 10^6^ cells and 2.5 × 10^7^ cells were incubated in 5 mL MM with 1% glucose and 1% arabinose, respectively. For the conditional mutant, 5 × 10^7^ and 2.5 × 10^7^ cells were incubated in 5 mL of MM with 1% glucose and 1% arabinose, respectively. This approach led to similar cell numbers after incubation for the different strains and the different carbon sources.

Cells for the low iron condition in regular MM and the high iron condition in MM with 50 μM FeCl_3_ in glucose and arabinose were prepared as described for the 5 h time point. For each condition, cells were harvested and RNA was extracted with the Qiagen RNA mini kit (Qiagen, Toronto, ON, Canada). A total of 500 ng of DnaseI-treated total RNA was used to prepare cDNA with the High-Capacity cDNA Reverse Transcription Kit (Thermo Fisher Scientific, Waltham, MA, USA). Quantitative PCR (qPCR) for selected genes (primers and genes listed in Table S1) plus one housekeeping gene (*syf1* UMAG_03842) was performed to confirm the expression changes seen in the RNA sequencing experiment, to establish gene expression patterns for the conditional mutant in the FB1 background, and to determine expression changes at different timepoints and in low and high iron environments. qPCR was performed as described before [20]. All results were normalized against the FB1 glucose condition or the FB1 high iron plus glucose condition.

For RNA-seq analysis, cells of the FB2 strain and corresponding *P_crg1_::grx4* strain were grown for 24 h and RNA was extracted as described previously [19]. DnaseI-treated total RNA was used for RNAseq, which was performed using Genewiz (South Plainfield, NJ, USA) according to their platform and protocols. Transcript levels were determined by mapping reads to the *U. maydis* genome Umaydis521_2.0 using STAR (version 2.7.9a) [21], and differential gene analysis was completed with the DESeq2 R package (version 1.10.1) [22]. To identify genes activated by Grx4, the genes were first sorted for significant changes (*p* < 0.05) between wt and mutant when grown in glucose and then all genes with a repression in the mutant of >2-fold were selected. Furthermore, the gene expression in arabinose should be unchanged (<2-fold change) or increased for those genes in the mutant. To search for genes repressed by Grx4, we first selected all genes with significant changes (*p* < 0.05) between wt and mutant in glucose and then selected all genes with an increased expression in the mutant of more than 2-fold. Second, these genes should show unchanged (<2-fold) or reduced expression for the mutant in arabinose. For genes which were activated or repressed by Grx4, a gene enrichment analysis with g:Profiler was performed (Table S3).

### 2.7. Siderophore Measurement

The chrome-azurol S (CAS), hexadecyltrimethylammonium bromide (HDTMA) assay was used to determine siderophore content in culture supernatants. The CAS-HDTMA assay solution was prepared as described in [23]. Supernatants of cultures (100 μL) after 72 h of growth were mixed with the CAS-HDTMA assay solution, incubated, and absorbance at 630 nm was determined. The absorbance was normalized against 10^6^ cells for each strain and expressed relative to FB1 grown in MM with 1% glucose.

### 2.8. Assays for Cellular Pigmentation and Extracellular Melanin

Conditional *P_crg1_::grx4* strains consistently showed visible pigment formation in MM plus 1% glucose in cultures without depletion of Grx4 or depleted cultures inoculated with a high cell number (1 × 10^7^ cells per mL) for 48 h or cultures incubated for extended incubation periods of 6 days. Extracellular melanin was measured in 100 µL of a cell-free supernatant for each strain at 405 nm in 96 well plates with a Tecan plate reader [24]. Furthermore, the change in pigmentation of the isolated cells of the different conditions was measured at OD_600_. OD_600_ of washed cells was measured for 200 uL for each strain with a cell suspension of 5 × 10 ^6^ cells per ml. The gene expression induction of *pks1* (4.9×), *lac1* (10.9×), and *mtf1* (61.2×) in cultures repressed for Grx4, together with the measurement of extracellular melanin, suggested that the cell associated pigment was related to melanin. 

### 2.9. Virulence

To assess the virulence of wt and *P_crg1_::grx4* strains, the cells in the two different mating backgrounds for wt and mutants were pre-grown after initial Grx4 depletion for 24 h in MM with 1% glucose, for 24 h in MM with 1% arabinose, or for 24 h in PDB with 1% arabinose with addition of 1% arabinose during plant infection, respectively. The cells were washed twice with water and mixed at a ratio of 1:1 at a final concentration of 5 × 10^6^ cells per ml. An amount of 50–100 μL of the cell suspension was injected into seven-day old seedlings of the maize variety Golden Bantam. After 14 d of infection, symptoms were scored. Approximately 100 plants were infected for each treatment in three independent experiments. Disease symptoms were scored as described previously [25,26].

### 2.10. Data Availability, Genomic and Bioinformatic Resources

The RNA-seq data were deposited in Gene Expression Omnibus under accession GSE239366. The genomic and bioinformatic resources were from FungiDB (https://fungidb.org/fungidb/app (accessed on 10 May 2023)), the Saccharomyces Genome Database (www.yeastgenome.org/ (accessed on 10 May 2023)), NCBI (www.ncbi.nlm.nih.gov/ (accessed on 10 May 2023)), g:Profiler (https://biit.cs.ut.ee/gprofiler/gost (accessed on 25 April 2023)), ClustalW (www.genome.jp/tools-bin/clustalw (accessed on 17 May 2023)), and aatbio (www.aatbio.com/ (accessed on 25 October 2023)).

### 2.11. Statistics

All experiments were repeated at least three times with three biological replicates. Values given are the average of triplicates ± SD. Data were analyzed with ANOVA plus Tukey’s, Kruskal Wallis plus Dunn’s, or a *t*-test for statistical significance. As pre-tests, Shapiro-Wilk and Levene tests were used. Data were generally compared with the respective control, and lines with significance indicators depicted in graphs indicate significant differences between those sample groups.

## 3. Results

### 3.1. Identification of the Grx4 Monothiol Glutaredoxin in U. maydis

The *S. cerevisiae* genome encodes eight glutaredoxin proteins which control various cellular functions, ranging from redox state maintenance and ROS protection to iron acquisition [27,28,29,30]. The protein sequences of these glutaredoxins were used as BLASTp queries to identify potential homologs in the *U. maydis* genome. Four genes encoding candidate Grx proteins were identified, including UMAG_04948, which corresponds to yeast Grx1 (2e^−27^)/Grx2 (5e^−23^)/Grx8 (2e^−6^), and UMAG_04223, which represents the sole Grx3 (2e^−55^)/Grx4 (4e^−63^) homologue. Furthermore, Grx4 protein from the basidiomycete *C. neoformans* was used to confirm the *U. maydis* homolog. The UMAG_02867 product was similar to yeast Grx5 (3e^−49^), and the UMAG_05435 product was similar to Grx6 (7e^−8^)/Grx7 (2e^−9^). 

**Figure 1:**
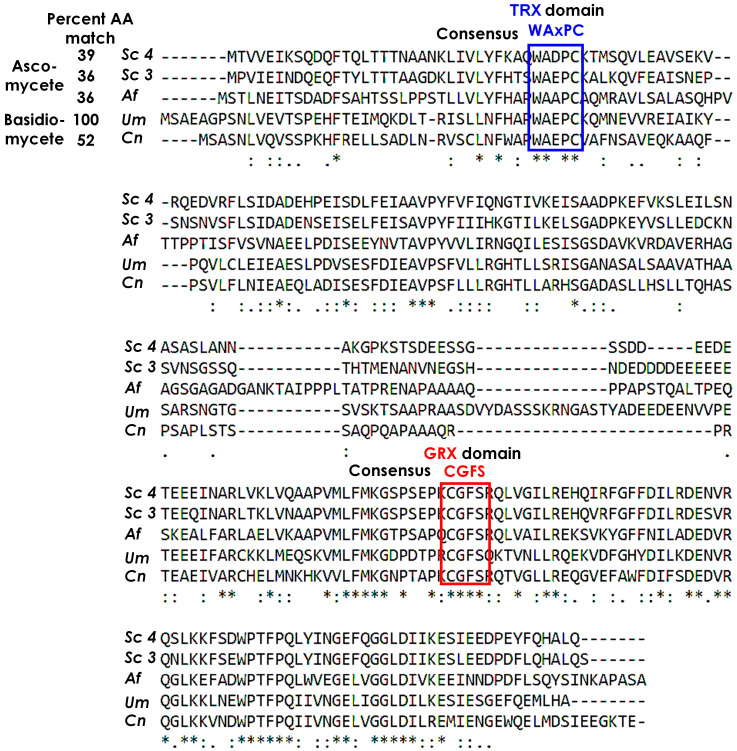
Alignment of different Grx3/4 amino acid sequences from *Saccharomyces cerevisiae* Grx3 (Sc 3) and Grx4 (Sc 4), *Aspergillus fumigatus* (Af), and *Cryptococcus neoformans* (Cn), with the putative *Ustilago maydis* Grx4 homologue UMAG_04223. The percentage of identical amino acids is relative to *U. maydis*. The identified consensus TRX domain has the sequence WAxPC and the GRX domain sequence is CGFS, which is typically found in Grx’s involved in iron metabolism. Cn: CNAG_02950, Af: Afu2g14960, and Um: UMAG_04223 were obtained from the fungiDB database. Sc: Grx3 and Grx4 were retrieved from the Saccharomyces Genome Database. Alignment was performed with ClustalW.

In addition to UMAG_04223, a BLASTp search using the yeast Grx4 protein sequence (YER174C) as a query identified *U. maydis* genes for Grx5 (UMAG_05435) and three thioredoxin proteins (UMAG_04579; UMAG_10953; UMAG_01370). The thioredoxins Trx1 and Trx2 in yeast are important for redox function as well as sulfur metabolism [31,32]. The TRX domain of *U. maydis* Grx4 (UMAG_04223) showed the conserved motif WAxPC when compared with the monothiol glutaredoxins of *C. neoformans*, *A. fumigatus,* and yeast, and all of the compared GRX domains contained the CGFS sequence typically found in monothiol glutaredoxin proteins regulating iron homeostasis (Figure 1). Overall, this analysis indicated that UMAG_04223 encodes the sole Grx4 homologue in *U. maydis* identifiable from existing, publicly available sequence data of strain 521. 

### 3.2. Growth of Strains with Conditional Grx4 Expression

**Figure 2:**
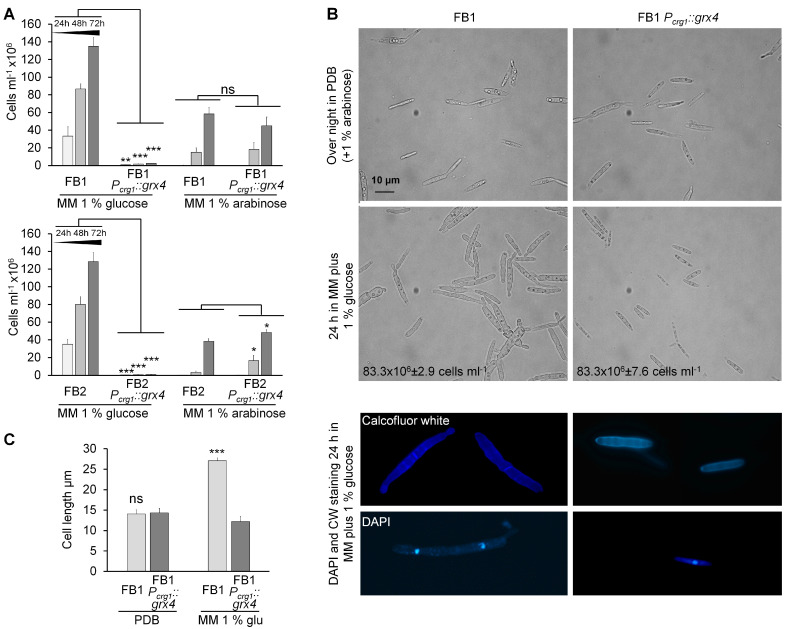
Cell shapes and growth rates of wt and *P_crg1_::grx4* strains during Grx4 depletion and post-depletion in repressing and activating conditions. (**A**) Shown are the cell numbers over time from 24 h to 72 h of FB1/FB2 wt and FB1/FB2 *P_crg1_::grx4* strains in MM with 1% glucose or 1% arabinose after prior Grx4 depletion. (**B**) Images of the cell shapes of wt (PDB) and *P_crg1_::grx4* (PDB + 1% arabinose) strains during overnight growth, and growth after 24 h of Grx4 depletion in MM with glucose. The cells pictured are representative of the morphologies observed in culture. Cells were stained with calcofluor white and DAPI as described in the Materials & Methods. (**C**) The quantification of cell sizes of wt and *P_crg1_::grx4* strains during growth in PDB overnight and after 24 h Grx4 depletion in MM with 1% glucose is shown. The average lengths of 25 cells for each biological repeat were obtained. Data are the average of three biological replicates with standard deviation. Significance levels are * < 0.05; ** < 0.01; *** < 0.001; ns: not significant. ANOVA plus Tukey’s or *t*-test was used to analyze the data for significant differences.

Attempts to delete or disrupt the *grx4* gene in *U. maydis* were unsuccessful, suggesting that Grx4 is essential for growth. Therefore, an alternative approach of expressing *grx4* from a conditional promoter was employed to test the essential nature of the gene and evaluate phenotypes associated with loss of Grx4. This strategy made use of the carbon-source regulated promoter (designated *P_crg1_)* from the *crg1* gene (*UMAG_03416*). Transcription of *crg1* is induced during growth in the presence of arabinose and repressed in the presence of glucose [16]. Two independent strains carrying the *P_crg1_::grx4* construct were generated for each wt background (FB1 and FB2) and subsequently characterized [19]. The strains within each background behaved similarly with regard to growth (Figure 2A). Specifically, cells of the conditional strains with the *P_crg1_::grx4* construct showed similar growth rates in MM with 1% arabinose compared with wt strains. Both wt strains demonstrated lower growth on MM with 1% arabinose than on MM with 1% glucose. Repression of *grx4* expression by 1% glucose in the *P_crg1_::grx4* strains markedly reduced growth compared with wt (Figure 2A). The *P_crg1_::grx4* strains grew similarly to wt on 1% arabinose after initial depletion, suggesting that reduced growth rates are not related to starvation-induced cell death during initial Grx4 depletion (Figure 2A). Overall, this analysis of the growth phenotypes supported the essentiality of Grx4 and established the conditions necessary to deplete Grx4 in preparation for subsequent studies.

The growth experiments revealed an influence of Grx4 on cell size. That is, cells of the wt strains pre-grown in PDB and the *P_crg1_::grx4* strain grown in PDB with 1% arabinose showed similar cell numbers, cell shapes, and cell sizes (Figure 2B,C). However, after 24 h of growth in MM with 1% glucose at a high inoculum of 2–3 × 10^8^ cells, wt cells showed an approximately 2-fold greater cell length compared with the cells of the *P_crg1_::grx4* strain depleted for Grx4 (Figure 2B,C). Interestingly, the cell numbers between the cultures were not significantly different. The larger wt cells were frequently multi-cellular with one nucleus per cell compartment according to calcofluor white and DAPI staining (Figure 2B). In contrast, cells of the *P_crg1_::grx4* strain after 24 h in MM with 1% glucose were comparable in size and shape to the initial wt cells grown in PDB or the *P_crg1_::grx4* cells grown in PDB plus 1% arabinose (Figure 2B,C). The results are shown for the strains of the FB1 background and similar phenotypes were observed for the FB2 strains. These observations indicated impaired cell separation by wt cells during growth at high inoculum in MM with glucose and that ordinary turnover of Grx4 may play a role in this process. In addition, *P_crg1_::grx4* cells grew normally in PDB medium (glucose) with 1% arabinose. indicating that glucose is not able to completely block *grx4* expression when both carbon sources are present.

**Figure 3:**
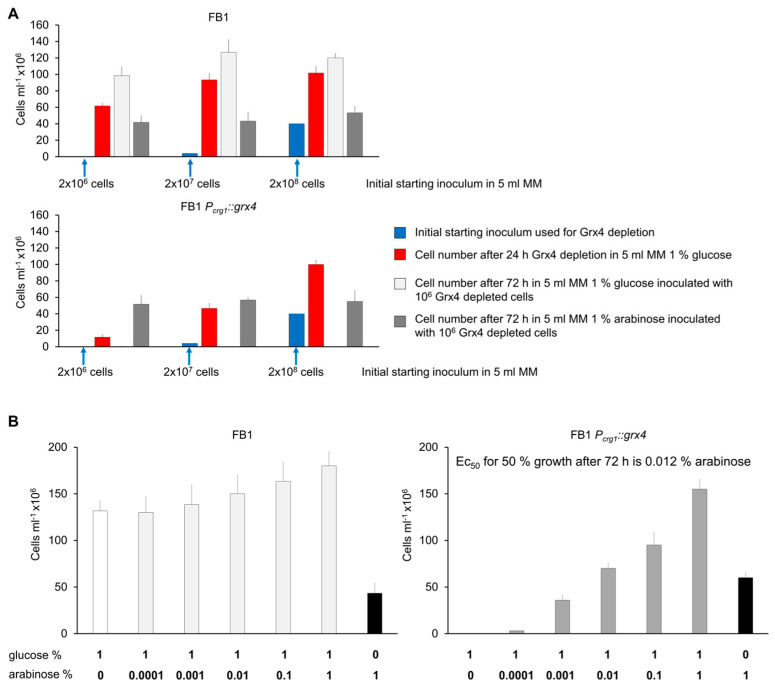
Establishment of Grx4 depletion conditions. (**A**) The growth rate during Grx4 depletion is dependent on inoculum size. Furthermore, the growth of mutant and wt in glucose or arabinose minimal medium for 72 h after initial Grx4 depletion is shown. For consistency, the same colors are used to indicate the inoculum (blue) and cell numbers at 24 h (red) for the FB1 and mutant strains, although the depletion of Grx4 refers to the mutant strain. (**B**) Growth of wt and the *P_crg1_::grx4* strain after Grx4 depletion in glucose with increasing amounts of arabinose. The EC_50_ value was calculated (www.aatbio.com/tools/ec50-calculator (accessed on 25 October 2023)) at 72 h. Data are the average of three biological replicates with standard deviation.

We also evaluated the influence of inoculum size on growth of the wt and conditional strain in glucose and arabinose to further establish the parameters for *grx4* depletion (Figure 3A). Two experiments are depicted in Figure 3A. First, different starting inoculum sizes (indicated in blue) were used to start cultures in 1% glucose for the FB1 wt strain and the *P_crg1_::grx4* mutant with subsequent measurements of growth at 24 h (indicated in red). This experiment revealed that lower inoculum levels led to limited growth of the mutant, indicating depletion of Grx4. The second experiment employed the lower inoculum level of Grx4 depleted cells to demonstrate that the mutant failed to grow after 72 h on glucose compared with substantial growth on arabinose. Additionally, a titration experiment with increasing concentrations of arabinose from 0 to 1% in MM with 1% glucose established that 0.0001% arabinose increased the *P_crg1_::grx4* cell numbers by ~12-fold at 72 h and that the EC_50_ to reach half the level of wt growth after 72 h was 0.012% arabinose under these conditions (Figure 3B). The cell number for the wt strain in 1% glucose plus 1% arabinose was 1.4-fold higher compared with 1% glucose alone. Taken together, we find that Grx4 is important for normal cell morphology, and that repressing and activating conditions after depletion can be employed with the *P_crg1_::grx4* strain for the analysis of Grx4-regulated functions.

### 3.3. Identification of Grx4-Regulated Gene Functions

After characterizing the strains with conditional *grx4* expression, we investigated the impact of blocking *grx4* expression on the transcriptome by performing RNA-seq analysis. The *P_crg1_::grx4* and wt strains were grown in MM either with glucose or arabinose for 24 h after initial Grx4 depletion. We predicted that transcript levels for genes positively influenced by Grx4 would be reduced due to the substantial repression of *grx4* in *P_crg1_::grx4* strains on glucose, and elevated or at least unchanged due to the increase in *grx4* expression upon growth on arabinose. The opposite pattern would be observed for the transcript levels of genes negatively influenced by Grx4. To identify genes with reduced transcript levels (indicating a positive influence of Grx4), we first sorted the genes for significant changes (*p* < 0.05) between the wt and the *P_crg1_::grx4* strain grown in glucose-containing media, and then selected all genes with a reduction of >2-fold. The transcript levels in cells grown in arabinose-containing media should be unchanged (<2-fold change) or increased for those genes in the conditional strain. This combined analysis identified 682 genes with transcript levels positively influenced by Grx4. To search for genes with elevated transcript levels, suggesting a negative influence of Grx4, we first selected all genes with significant changes (*p* < 0.05) between wt and the *P_crg1_::grx4* strain in glucose and then selected all genes with increased transcript levels of >2-fold in the conditional strain. These genes should show unchanged (<2-fold) or reduced transcript levels for the *P_crg1_::grx4* strain in arabinose. This combined analysis identified 807 genes as putatively regulated with Grx4 acting in a negative manner (Table S3).

Next, we performed a g:Profiler analysis (for Gene Ontology and Kegg terms) for the regulated transcripts in the positive and negative categories [33] (Table S3). As with the previous FunCat evaluation [19], this analysis revealed that Grx4 acts as a positive regulator of functions related to iron acquisition (GO:0019290, GO:0009237, GO:0006879, GO:0031169, GO:0031168, GO:0019539, GO:0010106), carboxylic acid metabolism (GO:0006082, GO:0019752, GO:0032787), and GSH metabolism (KEGG:00480). A closer inspection of predicted gene functions identified GSH metabolism, high-affinity iron uptake proteins, and siderophore functions as positively influenced by Grx4. As a negative regulator, Grx4 influenced transcript levels for iron binding functions (GO:0005506). Further inspection of predicted gene functions identified *pks1*, *lac1,* and *mtf1,* which are important for melanin formation, as negatively regulated by Grx4. Given the role of Grx4 in the regulation of iron homeostasis in other fungi [6], we focused our attention primarily on genes for iron-related functions (Table S3) for validation and follow-up experiments.

### 3.4. Expression of Grx4 and Iron-Regulated Genes at Different Timepoints and in Low and High Iron

**Figure 4:**
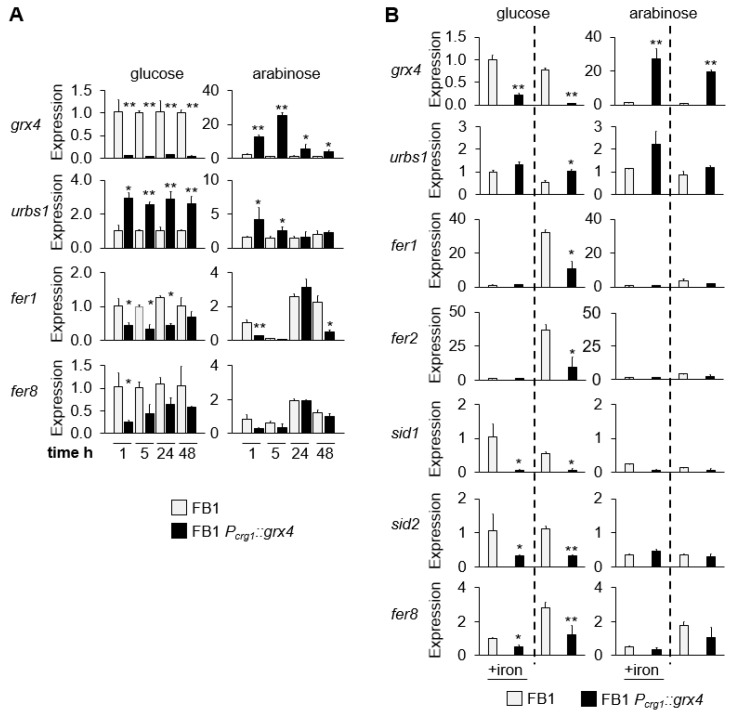
Expression of *grx4* and iron-related genes in FB1 and FB1 *P_crg1_::grx4*. (**A**) Time dependent expression of *grx4* and selected iron related genes (*urbs1, fer1, fer8*) over time for the time points of 1 h, 5 h, 24 h, and 48 h after transfer from the Grx4 depletion condition to MM with 1% glucose or 1% arabinose. (**B**) Expression of iron-related genes (*grx4, urbs1, fer1, fer2, sid1, sid2, fer8*) five hours after transfer from the Grx4 depletion condition to new MM with 1% glucose or 1% arabinose in low (0.185 μM) and high iron (50 μM). Data are the average of three biological replicates with standard deviation. Significance levels are * < 0.05; ** < 0.01. Data without significance markings are not significantly different between mutant and wt. ANOVA plus Tukey’s or *t*-test was used to analyze the data for significant differences.

We next performed a qPCR analysis to evaluate changes in transcript levels for *grx4* and iron-related genes at different times (1 h, 5 h, 24 h, and 48 h) with the goal of more closely examining repression and activation by glucose and arabinose in *P_crg1_::grx4* strains after depletion of Grx4 (Figure 4A). We specifically included the *urbs1* gene encoding the iron regulatory GATA factor along with the *fer1* gene encoding a multicopper oxidase of the high-affinity uptake system and the *fer8* gene encoding an iron-regulated gene of unknown function implicated in siderophore biosynthesis [13]. For comparison, we included an analysis of *grx4* expression in the same time course. After depletion, repression of *grx4* transcript levels by glucose was similar at the different time points. However, activation of *grx4* transcription by arabinose after 24 h of depletion was strongest at the early timepoints of 1 h and 5 h, and less markedly elevated at 24 h and 48 h. The transcript levels for target genes of Grx4 regulation (i.e., *urbs1*, *fer1,* and *fer8*) also showed the most robust differences at early timepoints (Figure 4A), and the 5 h time point was therefore selected for the subsequent analysis of the expression of a selected set of iron-regulated genes in low and high iron conditions (Figure 4B).

Our analysis of the response to iron revealed that the wt strain did not show significant regulation of *grx4* expression in low vs. high iron or in glucose vs. arabinose (Figure 4B). As expected, the *P_crg1_::grx4* strain showed strong repression of *grx4* in glucose. At 5 h, the magnitude of repression was greater in low iron conditions (32.3-fold) than in the high iron conditions (4.5-fold). In arabinose, the increased expression of *grx4* in the regulated strain differed only slightly between low and high iron conditions (19.3-fold vs. 27.4-fold, respectively). Transcript levels for Urbs1 were not markedly different in low vs. high iron or glucose vs. arabinose in the wt or the mutant. In the wt, genes encoding components of the high-affinity iron uptake system (*fer1, fer2*) were up-regulated in low iron medium with glucose at the early 5 h timepoint compared with high iron conditions. The genes encoding siderophore biosynthetic enzymes (*sid1*, *sid2*) and the *fer8* gene were mostly unchanged in expression in response to iron at this early timepoint for wt. In glucose at 5 h, the *P_crg1_::grx4* strain showed repression of transcript encoding components of both high-affinity iron uptake systems compared with wt. Furthermore, transcript encoding components of the siderophore uptake system were repressed in both the high and low iron conditions, while *fer1* and *fer2* were only repressed in the low iron condition compared with wt. In arabinose, the conditional strain showed similar regulation of all Grx4 target genes compared with wt (Figure 4B). Together, these results support the involvement of Grx4 in regulation of the known iron acquisition systems in *U. maydis*.

### 3.5. Addition of Iron Partially Restores Growth in the Context of Grx4 Depletion

**Figure 5:**
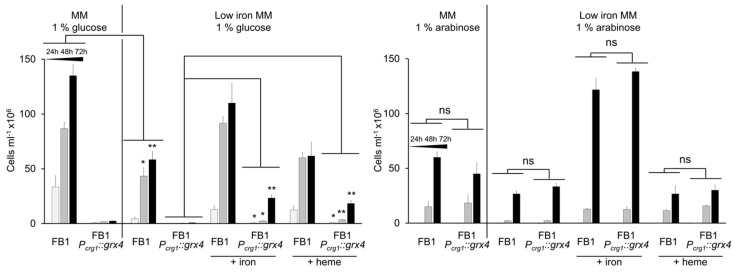
Addition of iron partially rescues growth of the *P_crg1_::grx4* strain on glucose. Growth over time of wt and the *P_crg1_::grx4* strain on glucose or arabinose in a low iron environment after 24 h iron starvation compared with growth in regular minimal medium without iron pre-starvation. Additionally, the growth of wt and mutant in low iron with addition of 50 μM FeCl_3_ or 30 μM heme is shown. Data are the average of three biological replicates with standard deviation. Significance levels are * < 0.05; ** < 0.01; ns: not significant. ANOVA plus Tukey’s or *t*-test was used to analyze the data for significant differences.

As described above, genes for iron acquisition were regulated by Grx4. We thus tested whether exogenous supplementation of iron could suppress the growth defect of the *P_crg1_::grx4* strain under *grx4*-repressing conditions. The results were compared with the wt strain grown in glucose or arabinose, and the conditional strain grown in arabinose when Grx4 was expressed. Pre-starvation for iron for 24 h reduced the subsequent growth of wt and conditional strains in low iron MM with glucose or arabinose as the carbon source compared with regular MM (Figure 5). Addition of heme or FeCl_3_ increased the growth of the *P_crg1_::grx4* strain (at 48 h and 72 h, respectively) in MM with glucose and thus partially rescued the growth defect of the conditional strain in the low iron environment. In MM with arabinose, wt and the *P_crg1_::grx4* strain showed similar growth rates in the different media with different iron levels (Figure 5). 

### 3.6. Siderophore Formation Is Regulated by Grx4

**Figure 6:**
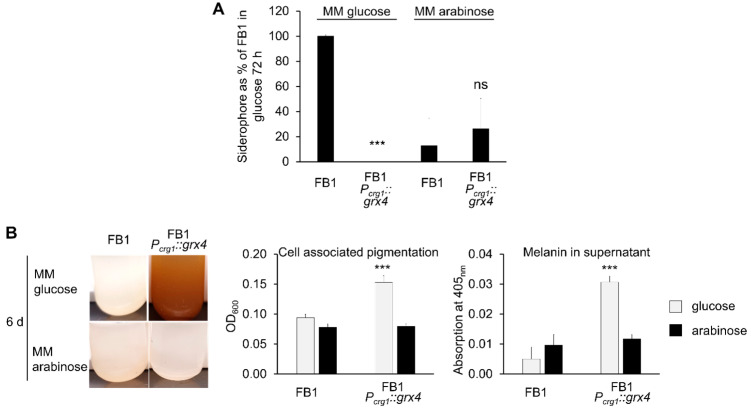
Grx4 repression abolishes siderophore formation and provokes pigment/melanin formation. (**A**) Siderophore production after 72 h of growth in MM with 1% glucose or 1% arabinose by FB1 and the FB1 *P_crg1_::grx4* strains after initial Grx4 depletion. Siderophore levels were normalized according to the cell numbers in the respective cultures and were expressed relative to FB1 grown in MM with glucose. (**B**) Culture phenotypes of FB1 and the FB1 *P_crg1_::grx4* strain without Grx4 depletion grown for 6 d in MM with 1% glucose or 1% arabinose are shown. Cell associated pigmentation was assessed by OD_600_ for the same number of washed cells of FB1 and the FB1 *P_crg1_::grx4* strains. Extracellular melanin was measured by absorbance at 405 nm for the supernatant of cultures for the FB1 and FB1 *P_crg1_::grx4* strains. Data are the average of three biological replicates with standard deviation. Significance levels are *** < 0.001; ns: not significant. ANOVA plus Tukey’s was used to analyze the data for significant differences.

The *P_crg1_::grx4* strain grown on MM with glucose showed reduced transcript levels for the *sid1*, *sid2,* and *fer8* genes, and for iron acquisition genes in general (Table S3, Figure 4). On arabinose, the transcript levels for the genes in the conditional strain were similar to wt. These observations prompted a measurement of siderophore activity after three days of growth in MM with glucose. After adjustment for cell numbers, siderophore activity was readily detectable in the wt but not in the conditional strain (Figure 6A). Siderophore activity was similar between wt and conditional strains when grown with arabinose as the carbon source (Figure 6A). We also noted that the carbon source influenced siderophore activity, with reduced activity on arabinose compared with glucose. This observation may indicate a greater demand for iron for cells grown in glucose. Overall, this phenotypic analysis was therefore consistent with the transcript data and indicated that siderophore production is regulated by Grx4.

### 3.7. Pigment/Melanin Formation Is Repressed by Grx4

The transcriptome data indicated increased transcript levels of genes for all known melanin biosynthesis pathways in *U. maydis,* consisting of *pks1*, *lac1*, and *mtf1* in the *P_crg1_::grx4* strain when grown in glucose [19] (Table S3). The analysis of melanin formation is challenged by the severely reduced growth of the conditional strain on glucose after Grx4 depletion. We addressed this challenge by inoculation with a higher cell number for shorter incubation times or incubation for a longer time of six days without prior Grx4 depletion and found pigment formation in the cultures (Figure 6B). A measurement of extracellular melanin indicated a 6.1-fold increase for the *P_crg1_::grx4* glucose supernatant. Pigments associated with washed cells increased the OD_600_ 1.6-fold for the cells of the conditional strain compared with wt (Figure 6B). Similar absorbances were found for both strains when grown on arabinose (Figure 6B). Overall, these results support the conclusion that Grx4 is a negative regulator of melanin formation in *U. maydis*.

### 3.8. Appropriate Grx4 Levels Are Important for Mating

**Figure 7:**
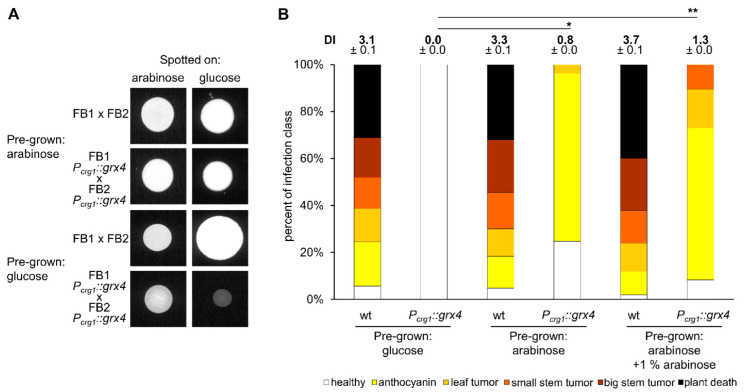
Grx4 repression reduces mating and abolishes virulence. (**A**) Mating reactions of FB1 × FB2 and FB1- *P_crg1_::grx4* × FB2- *P_crg1_::grx4* strains after 48 h on DCM with 1% glucose or 1% arabinose with (pre-grown in glucose) and without (pre-grown in arabinose) Grx4 pre-depletion. (**B**) Virulence of wt (FB1 × FB2) and *P_crg1_::grx4* strains (FB1- *P_crg1_::grx4* × FB2- *P_crg1_::grx4*) pre-grown in glucose, pre-grown in arabinose or pre-grown in PDB with arabinose plus addition of 1% arabinose during plant infection are shown. An amount of 1% arabinose in water alone when injected into the seedlings did not show plant toxicity. Data are the average of three biological replicates with standard deviation. Significance levels are * < 0.05; ** < 0.01. Kruskal-Wallis and Dunn’s test was used to analyze the data for significant differences.

To assess the mating ability of the wt and conditional *P_crg1_::grx4* strains, cells in both mating type backgrounds (FB1 and FB2) were pre-grown in MM with either glucose or arabinose, washed, mixed at a ratio of 1:1, and then transferred to mating assay plates (DCM+ activated charcoal) with either glucose or arabinose as the carbon source. Pre-grown cells in arabinose-containing media for mixtures of wt (FB1xFB2) or the *P_crg1_::grx4* strains (FB1 *P_crg1_::grx4* x FB2 *P_crg1_::grx4*) cells did not show any mating deficiencies independent of the carbon source in the mating agar (Figure 7A). Thus, mating was not perturbed in the *P_crg1_::grx4* strains under activating or repressing conditions when pre-grown in the activating condition. In contrast, cells of the *P_crg1_::grx4* strains in both mating backgrounds grown in glucose failed to produce a strong mating reaction when transferred to DCM with glucose and had a reduced mating reaction when transferred to DCM containing arabinose (Figure 7A). A mixture of the wt strains mated normally on DCM plates with either glucose or arabinose. These results indicated that mating of the *P_crg1_::grx4* strains after Grx4 depletion is severely restricted on glucose, and even in the arabinose activating condition.

### 3.9. Grx4 Is Needed for Virulence and Pathogenic Development

To assess the virulence of the wt and the conditional *P_crg1_::grx4* strains, cells in both mating backgrounds were pre-grown for 24 h in MM with either glucose or arabinose and used to infect maize seedlings. Plants inoculated with the *P_crg1_::grx4* cells pre-grown in glucose failed to develop any disease symptoms, while infections with wt strains showed the typical range of smut disease symptoms (Figure 7B). Plants inoculated with the *P_crg1_::grx4* cells pre-grown in arabinose developed early disease symptoms, such as anthocyanin formation, but failed to develop later symptoms, including tumor formation. The wt strains grown on arabinose caused typical disease symptoms similar to wt cells pre-grown in glucose. To test if addition of arabinose improved virulence of the conditional strains, the *P_crg1_::grx4* cells pre-grown in arabinose were employed with the addition of 1% arabinose to cells (wt and *P_crg1_::grx4* strains) during inoculation of the plants. Arabinose did not have any negative impact on maize when injected in the absence of fungal cells. However, addition of arabinose increased the virulence of the *P_crg1_::grx4* strain such that the strains caused small tumors on leaves and the stems of seedlings, although the full range of disease symptoms was not achieved (Figure 7B). This result indicates that the expression level of Grx4 is a key determinant for symptom formation, and supports the conclusion that Grx4 contributes to virulence. An analysis with the solopathogenic strain SG200 that does not require mating for initiating an infection showed a similar reduction in virulence [19]. Overall, these results suggest that Grx4 is required for the virulence of *U. maydis*. 

## 4. Discussion

Monothiol glutaredoxins such as Grx4 are key regulators of a wide variety of cellular processes in fungi, including iron uptake and homeostasis, iron sulfur cluster maturation and trafficking, maintenance of the cellular redox balance, the response to DNA damage, vacuolar organization, and cell wall integrity [6,7,8,9,10,11,34,35,36,37]. Given our previous demonstration of the importance of Grx4 in iron homeostasis and the virulence of *C. neoformans* [9], we embarked on an analysis of the role of the protein in another basidiomycete pathogen, *U. maydis*. We initially failed to disrupt or delete the *grx4* coding sequence which suggested that the gene is essential. There is precedent for this conclusion because full deletion of the *grx4* gene in *C. neoformans* was also unsuccessful, leading to a gene disruption approach [9]. Additionally, GrxD in *A. fumigatus* is also essential [10]. In the latter case, carbon source-regulated alleles were employed to study Grx4 function, and we adopted a similar approach to study the protein in *U. maydis*. 

In our study, we initially characterized the expression pattern for the *grx4* transcript produced from the arabinose inducible, glucose repressible promoter of *crg1* to establish the timing and control of *grx4* expression for subsequent experiments. We then employed RNAseq and qPCR to obtain an overview of the impact of depletion of Grx4, and we found the anticipated regulation of iron homeostasis functions in low and high iron environments. These results are consistent with the role of monothiol glutaredoxins in interactions with iron regulatory proteins in the model yeasts *S. cerevisiae* and *S. pombe*, as well as in fungal human pathogens, including *A. fumigatus*, *C. albicans,* and *C. neoformans* [7,8,9,10,11,36,38]. We specifically found that iron-regulated genes, including those encoding the high-affinity uptake system and siderophore biosynthesis, were differentially regulated upon depletion of Grx4. 

Our phenotypic characterization of the roles of Grx4 using the conditional strains revealed regulation of a number of phenotypes related to virulence. These included the formation of melanin, a pigment that accumulates in developing teliospores during infection, and reduced mating reactions when Grx4 was depleted. Importantly, the conditional strains allowed us to test the relevance of Grx4 for disease symptom development by *U. maydis* in maize seedlings, and we found that disease was severely attenuated. This result supports a role for Grx4 in virulence, although it is possible that arabinose availability in maize seedlings was not sufficient or accessible to support expression of *grx4* from the *P_crg1_::grx4* construct. This was also confirmed by the slight increase in virulence when external arabinose was added during infection. However, it is also possible that a precise level of *grx4* expression is necessary for full symptom development. Our previous studies in *C. neoformans* also revealed a role for Grx4 in disease outcome in a murine model of cryptococcosis [9,11]. Furthermore, we found additional parallels between the results for Grx4 in *U. maydis* and *C. neoformans,* which include the influence on iron homeostasis and melanin formation. 

The essentiality of Grx4 in *U. maydis* is interesting in light of studies in yeast. Specifically, Grx3/4 paralogs in *S. cerevisiae* are known to be essential in strain W303, but not in strain BY4742 [39]. However, double mutants (*grx4*Δ *grx3*Δ) in the BY4742 strain are only viable in a low oxygen environment. After transfer to ambient air, these cells are prone to developing compensatory mutations [34]. Interestingly, there is increasing evidence that Grx4 is also connected to oxygen sensing and regulation via sterol binding transcription factors such as SrbA and the transcription factor AtrR in *A. fumigatus* [35]. In *U. maydis*, we previously showed that cultures with the combination of glucose and malate form melanin and are hypoxic, and we determined that tumor tissues in leaves and stems during biotrophy are also reduced in oxygen levels [5]. It is tempting to speculate that oxygen sensing via an unknow transcription factor together with Grx4/Urbs1 regulates iron homeostasis, mitochondrial functions (electron transport and the TCA cycle), ergosterol synthesis, and heme-related functions, and integrates central metabolism with the mating pathway to influence virulence and biotrophy. 

In summary we identified and characterized Grx4 in *U. maydis* and showed conserved iron regulon activation by the protein as well as an impact on melanin and virulence. This analysis provides a foundation for further analysis of the role of iron-regulatory factors in fungal pathogenesis.

## Data Availability

The RNA-seq data were deposited in Gene Expression Omnibus under accession GSE239366.

## Supplementary Materials

The following supporting information can be downloaded at: https://www.mdpi.com/article/10.3390/jof9111112/s1, Table S1: List of primer sequences; Table S2: *Ustilago maydis* strains used in this study; Table S3: RNA sequencing results and gene enrichment analyses for Grx4 regulated genes. Reference [40] is cited in the supplementary materials.
