# Supplementary material for: The Monothiol Glutaredoxin Grx4 Influences Iron Homeostasis and Virulence in Ustilago maydis
Source: J Fungi (Basel). 2023 Nov 17;9(11):1112. doi: 10.3390/jof9111112 (PMC10672361; doi:10.3390/jof9111112)
Supplement: Supplementary file 1 [file jof-09-01112-s001.zip › jof-2699854-SM.pdf]

Supplemental Tables

Table S1: List of primer sequences

Primer name	Sequence	Comments
276	CTCGGCCACTCAGGCCCAATGACAACACCGTTCCAG	*Grx4* coding sequence amplification
277	AAAAGATACCATAATAATGTCTGCCGAAGCAGGACCCA	
280	ATTGTCACGCCATGGTTGTTTGTTGGGACAATTAGTGGAAG	*Grx4* upstream sequence amplification
281	GAGGCCTAGATGGCCCTCTCGTGCGCAAAAGTGTGATG	
282	CTTTTGCGCACGAGAGGGCCATCTAGGCCTCGAGG	pMF2-1n amplification
279	AACGGTGTTGTCATTGGGCCTGAGTGGCCGAGCT	
283	ATTGTCCCAACAAACAACCATGGCGTGACAATTGCG	Amplification of the nourseothricin resistance cassette including the arabinose-inducible promoter P_{crg1}
278	CTGCTTCGGCAGACATTATTATGGTATCTTTTTGGCCTCA	
284	CAATGACAACACCGTTCCAGC	P_{crg1}::*grx4* final construct amplification
285	CTCTCGTGCGCAAAAGTGTG	
Umgrx4_frt	TTGTGCCCGAGACAGAAGAG	RT primers UMAG_04223
Umgrx4_rrt	CGTAGTGGCCGAAATCAACC	
Umurbs1_frt	AGGACGATGACCACTGGAACTG	RT primers UMAG_01050
Umurbs1_rrt	TCGGCGTAGAAGAGGACTCC	
Umsyf1_frt	CATTGCTGCTTCGGCTCTGG	RT primers UMAG_03842
Umsyf1_rrt	GGTGCTGGCTGCAACGAATG	
Umfer1_frt	CGGCTTACTTGCGCTTCAGG	RT primers UMAG_00105
Umfer1_rrt	CGTTGCCGGTGGTAGCAATC	
Umfer2_frt	GGAACAACACCGCCACCTAC	RT primers UMAG_10023
Umfer2_rrt	CTGCCTGGCCTGGATTGAAC	
Umfer8_frt	CTTTGTGATTGGCGATGCTG	RT primers UMAG_11338
Umfer8_rrt	TTGATGGCTGGTGGTGTTG	
Umsid1_frt	CGACTTCTTCCCAGCAAACG	RT primers UMAG_10188
Umsid1_rrt	TTGATGCGGTTCCTCTCCTC	
Umsid2_frt	CTCGATTACTCGCACCACTC	RT primers UMAG_10189
Umsid2_rrt	CACTAGCGTGTGCAGATCAG	

Table S2: *Ustilago maydis* strains used in this study

Strain	Background	resistance	Comments	source
FB1	Wt *a1b1*	None		[40]
FB2	Wt *a2b2*	None		[40]
P_{crg1}::*grx4* 52	FB1	Nat	Grx4 regulated strain. Glucose repression, arabinose activation	This study
P_{crg1}::*grx4* 53	FB1	Nat	Grx4 regulated strain. Glucose repression, arabinose activation	This study
P_{crg1}::*grx4* 55	FB2	Nat	Grx4 regulated strain. Glucose repression, arabinose activation	This study
P_{crg1}::*grx4* 56	FB2	Nat	Grx4 regulated strain. Glucose repression, arabinose activation	This study

Table S3 (Excel file): RNA sequencing results and gene enrichment analyses for Grx4 regulated genes.
